# Sudden restructuring of memory representations in recurrent neural networks with repeated stimulus presentations

**DOI:** 10.3389/fncom.2025.1601641

**Published:** 2025-10-22

**Authors:** Jonathon R. Howlett

**Affiliations:** ^1^VA San Diego Healthcare System, San Diego, CA, United States; ^2^Department of Psychiatry, University of California, San Diego, La Jolla, CA, United States

**Keywords:** Amari-Hopfield network, attractor dynamics, associative learning, discontinuous learning, Hebbian learning, nonlinear dynamics, computational modeling

## Abstract

While acquisition curves in human learning averaged at the group level display smooth, gradual changes in performance, individual learning curves across cognitive domains reveal sudden, discontinuous jumps in performance. Similar thresholding effects are a hallmark of a range of nonlinear systems which can be explored using simple, abstract models. Here, I investigate discontinuous changes in learning performance using Amari-Hopfield networks with Hebbian learning rules which are repeatedly exposed to a single stimulus. Simulations reveal that the attractor basin size for a target stimulus increases in discrete jumps rather than gradual changes with repeated stimulus exposure. The distribution of the size of these positive jumps in basin size is best approximated by a lognormal distribution, suggesting that the distribution is heavy-tailed. Examination of the transition graph structure for networks before and after basin size changes reveals that newly acquired states are often organized into hierarchically branching tree structures, and that the distribution of branch sizes is best approximated by a power law distribution. The findings suggest that even simple nonlinear network models of associative learning exhibit discontinuous changes in performance with repeated learning which mirror behavioral results observed in humans. Future work can investigate similar mechanisms in more biologically detailed network models, potentially offering insight into the network mechanisms of learning with repeated exposure or practice.

## Introduction

The study of learning is a central topic in psychology, cognitive science, and cognitive neuroscience. While analysis of average performance at the group level tends to suggest that learning is a smooth, incremental process (Thurstone, 1919; Newell and Rosenbloom, 1993), examination of individual learning curves often reveals sudden, discontinuous changes. For example, conditioning acquisition in individuals is typically characterized by abrupt transitions from low levels of responding to an asymptotic level (Gallistel et al., 2004). Similarly, while group average improvement in cognitive skills with practice can be approximated by a smooth power law function, individual performance exhibits plateaus punctuated by sudden improvements (Donner and Hardy, 2015). Discontinuities in performance also characterize language learning (van Dijk and Geert, 2007), the development of sudden cognitive insight (Smith and Kounios, 1996), and repetition learning in declarative memory (Musfeld et al., 2023).

Large changes in behavior in response to small changes in input are a hallmark of nonlinear systems. Threshold effects, in which smooth responses suddenly give way to dramatic shifts, are observed in simple nonlinear thermodynamic systems (Onuki, 2002) as well as highly complex systems such as climate (Rial et al., 2004) and ecosystems (Scheffer et al., 2001). Relatedly, nonlinear generating processes are frequently characterized by heavy-tailed distributions (Albert and Barabási, 2002). This implies that extreme values occur much more frequently than would be expected for Gaussian distributions, which are often generated by additive processes in linear systems (Roberts et al., 2015).

In neuroscience, nonlinear features of neural networks, such as nonlinear neural activation functions, are believed to play a critical role in supporting computational processes such as learning (Dayan and Abbott, 2005). One of the simplest neural network models of associative learning is the Amari-Hopfield network, a simple version of a recurrent neural network (RNN). Traditional Amari-Hopfield networks are fully connected single-layer networks consisting of binary neurons with simple rules for network evolution inspired by the Ising model of spin glasses in statistical mechanics (Hopfield, 1982; Amari, 1972; Amari, 1977). Using simple Hebbian associative learning rules, Amari-Hopfield networks can be trained to form attractors for remembered inputs, which cause similar states (within the attractor basin) to converge on these remembered states. Despite their simplicity, Amari-Hopfield networks have had major impacts on statistical mechanics, neuroscience, and machine learning, and modified versions of Amari-Hopfield networks with greater storage capacity continue to be investigated (Krotov, 2023).

While the storage capacity of traditional and modified Amari-Hopfield networks has been extensively studied, the effect of repeat stimulus presentations (or, alternatively, of increasing weight for a given input stimulus) has been less explored. Studying changes in attractor basins in RNNs with repeat stimulus presentations may be relevant to a range of cognitive phenomena, such as the effect of repetition training on memory performance (Musfeld et al., 2023) and the effect of practice on skill development [which may involve retrieval of stored procedural memories (Logan, 1988)].

In this brief report, I demonstrate that repeated stimulus presentations in synchronously updating Amari-Hopfield networks with a simple Hebbian learning rule cause intermittent, abrupt jumps in the size of attractor basins, rather than smooth incremental growth. Furthermore, I show that the distribution of positive jumps in attractor basin size is best approximated by a lognormal distribution, suggesting that the distribution is heavy-tailed. Examination of transition graphs before and after jumps in basin size reveals that newly acquired states tend to be organized into branching structures, suggesting that the heavy-tailed distribution of jumps in basin size may be related to the heavy-tailed distribution of branch sizes in the transition graph structure. The results suggest that even simple nonlinear models of associative memory exhibit characteristics of discontinuous learning and may have implications for more detailed, biologically realistic models of learning in neural networks.

## Methods

### Attractor basin changes with repeated stimulus presentations

Memory storage was modeled with an Amari-Hopfield network consisting of 10 binary neurons (± states). Small networks were studied to enable full enumeration of the state space (1,024 states for a 10-neuron vector). The network weights *W* were constructed using a batch Hebbian learning rule. Given a set of *p* stored patterns {*x*_1_*, x*_2_*, …, x_p_*}, each of length 10, the weight matrix was computed as:


$$W=X^{T}\;X,$$

Where *X* is the *p* × 10 matrix whose *μ*th row is the pattern *x_μ_*. The diagonal elements were set to zero (i.e., *W_ii_* = 0) to eliminate self-feedback.

In the experimental paradigm, the network was first “pretrained” with a diverse set of 50 random stimuli. To amplify the influence of these stimuli (which would later gradually be overcome by repeated presentation of the target stimulus), each of these 50 patterns was multiplied by 10, then combined into a 50 × 10 matrix which was used to produce initial network weights via the Hebbian learning rule. This pretraining phase established an initial attractor landscape with a rich set of stored memories with which the target stimulus would compete to attract states to its attractor basin.

The target stimulus was then generated randomly. In the target presentation phase, the network was presented repeatedly with the target stimulus, with the number of target presentations being incremented one at a time from 0 to 1,000. For each target presentation count *j* ∈ {1, 2, …,1,000}, the complete memory matrix was *X* was constructed by concatenating the pretraining matrix (of size 50 × 10) and *j* copies of the target stimulus. The weight matrix was then recomputed using the Hebbian learning rule.

The network dynamics were simulated using synchronous updates:


s(t+1)=sign(Ws(t)),

Where sign (·) returns 1 for nonnegative inputs and −1 for negative inputs. Convergence was determined when the state no longer changed between updates.

At each step during the target presentation phase, the attractor basin size was computed corresponding to the target pattern—that is, the number of initial states (sampled exhaustively) that converged to the target attractor. These basin sizes were stored and used to compute increases (or jumps) in attractor basin size during the target presentation phase.

Simulations were implemented in R (R Core Team, 2013). The experiment was repeated over 100 runs with new random pretraining stimuli and target stimulus being generated for each run.

### Distribution of basin size jumps

The basin size trajectories for the target stimulus were aggregated across runs to analyze the distribution of positive jump sizes in the target attractor basin. Jump size for stimulus presentation *j* was defined as the number of initial states that converged on the target state after stimulus presentation *j* minus the number of initial states that converged on the target state after stimulus presentation *j* − 1. Discrete jumps were defined as positive changes in basin size given that most stimulus presentations did not lead to a change in basin size. Excess kurtosis for the empirical distribution of positive jump sizes was estimated using the *e1071* package in R. Several candidate distributions were fit to the observed positive jump sizes using maximum likelihood estimation implemented in the R package *fitdistrplus*. The candidate models included a lognormal distribution, an exponential distribution, a half-normal distribution (centered at 1), and a power law distribution (with lower bound *x*_min_ = 1 and with the scaling exponent *α* estimated via the R package *poweRlaw*). Akaike information criterion (AIC) was computed for each distribution fit and cumulative distribution function (CDF) on a linear scale and complementary cumulative distribution function (CCDF) on a log scale were plotted for the empirical data and the fitted distributions. Parameter stability estimates for distributions were assessed using nonparametric bootstrapping (resampling 1,000 bootstrap replicates from observed data with replacement).

### Transition graph structure after basin size increase

In order to investigate the structure of memory representations after a basin size jump, the state transition graph immediately after each increase in basin size was examined. After an increase in basin size, the set of new states was defined as states belonging to the target basin after the jump but not belonging to the target basin before the jump. Transitions were based on single updates from one state to the next. Transition graphs were then constructed where each node represented a state of the network and a directed edge connected each state to the state obtained after one synchronized update. This yielded a branching tree structure in which each state’s “parent” was defined as the next state obtained after a synchronous update, and the root node was the target attractor state. To quantify the graph structure of the new states added after an increase in basin size, these new states were sorted into branches, and the size of each branch was recorded. To accomplish this, a set of branch heads was first identified within the new states, defined as new states whose immediate parent was not a new state (i.e., which had belonged to the attractor basin prior to the jump). For each of these branch head states, the number of its descendants were computed (while also counting the branch head state itself), thus quantifying the size of each branch of new states which had been added to the target basin. This procedure partitioned all new states into branches, with the sum of branch sizes being equal to the total number of new states after an increase in basin size. Branch sizes were defined as the number of states that eventually flow into a node in the state-transition graph. They were measured by exhaustively determining the descendent state of each possible network state.”

A network with 10 neurons was pretrained with 50 random patterns, each being multiplied by 10, and then a randomly selected target stimulus was presented 1,000 times, as in the previous experiments. Transition graphs were examined after each increase in target basin size, and branch sizes of new states were recorded. This process was repeated over 100 runs, with new random pretraining stimuli and target stimulus being generated for each run. The distribution of branch sizes for new states was then examined and fit with candidate distributions including a lognormal distribution, an exponential distribution, a half-normal distribution, and a power law distribution, as in the previous experiment.

For comparison purposes, the distribution of branch sizes was also examined for all states across a network (i.e., the total number of descendant states, also counting the state itself, for all states across a network). This was performed for a network with 10 neurons that had been pretrained with 50 random patterns, each being multiplied by 10. For visualization, the graph structure of a target attractor basin immediately before and after a basin size jump was plotted for a smaller network of 8 neurons.

### Sampled initial states in larger networks

In order to examine basin growth in larger networks, networks with 100 and 1,000 neurons were examined. Because it is not feasible to enumerate all possible starting states in networks of this size, starting states were randomly sampled from subsets of states with differing Hamming distances (i.e., number of flipped bits) k from the target stimulus. Distances k of 1, 2, 3, 5, 10, and 20 were sampled for the 100-neuron network and distances 1, 2, 3, 5, 10, 20, 50, and 100 were sampled for the 1,000-neuron network. For each distance k, 100 states of distance k from the target stimulus were randomly sampled. As in previous experiments, the networks were pretrained with random stimuli (one stimulus multiplied by weight 30 for the 100-neuron network and one stimulus multiplied by a weight of 100 for the 1,000-neuron network). As before, the target stimulus was repeatedly presented to the network (the target was presented 500 times). For each presentation of the target, the proportion of sampled initial states at each distance k which converged on the target (i.e., were in the target stimulus basin) was recorded. The same sampled starting states were used across stimulus presentations to avoid noise caused by resampling starting states.

### Effect of interference and stimulus degradation on jump distribution

Simulations were performed to investigate the effect of varying levels of memory interference (pretraining stimulus presentations) and stimulus degradation (distance k of the probe stimulus from the target stimulus) on the distribution of sizes of jumps in sampled initial states which are in the target stimulus basin. Simulations were performed on a network with 100 neurons. In order to examine the effect of pretraining stimulus presentations, k was held constant at 20 and simulations were performed with 1, 2, 3, 4, and 5 pretraining stimuli (each multiplied by a weight of 20). In order to examine the effect of k, the number of pretraining stimuli was held constant at 1 (multiplied by a weight of 20), and simulations were run with k of 5, 10, 15, 20, and 25. For each run, the target stimulus was presented 300 times. For each condition, 100 runs were performed. All jump sizes (with jumps defined as any increase in number of sampled starting states which converged on the target) were recorded, and the coefficient of variation of the distribution of positive jump sizes was calculated for each run. Welch’s ANOVA tests were performed to determine whether stimulus degradation (k) or memory interference (number of pretraining memories) significantly affected the coefficient of variation of jump sizes.

## Results

### Attractor basin changes with repeated stimulus presentations

**Figure 1:**
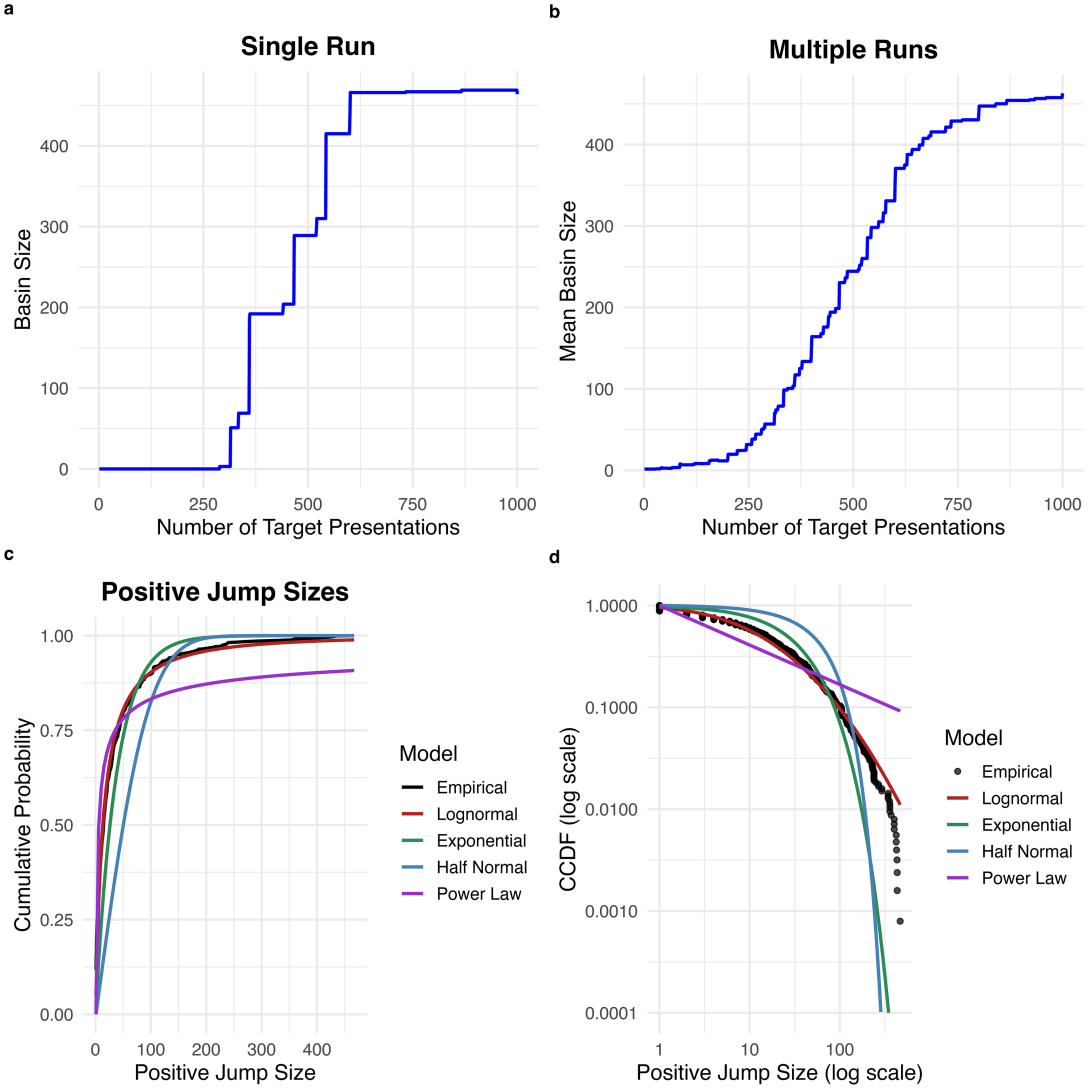
Discrete jumps in attractor basin size with repeated stimulus exposures. **(a)** Increase in basin size with repeated exposure for a single run. The basin size for the target stimulus increases in intermittent, discrete jumps. **(b)** Average increase in basin size over 100 runs. There is a smoother increase in basin size when averaging across runs. **(c)** Cumulative probability distribution for the size of positive jumps. The empirical distribution is best fit by a lognormal distribution. **(d)** Complementary cumulative distribution function (CCDF) for positive jump sizes plotted on a log scale.

Examination of plots of target attractor basin size with repeated target stimulus presentations revealed that attractor basin size increases in intermittent, abrupt jumps, rather than gradually and smoothly increasing (see Figure 1a for an example plot for one experimental run). The proportion of all stimulus presentations which caused an increase in attractor basin size was 0.0126. See Figure 1b for a plot of average increase in target basin size across experimental runs, which shows a relatively smooth increase with repeated stimulus presentations.

### Distribution of basin size jumps

Estimated excess kurtosis for the distribution of all positive jump sizes was 11.9, greater than the expected excess kurtosis for a half-normal distribution (~0.87) and for an exponential distribution (Smith and Kounios, 1996). This suggests that the distribution of positive jump sizes is heavy-tailed.

Based on AIC, the best-fitting distribution for positive jump sizes was the lognormal distribution (mean = 2.58, 95% CI [2.49, 2.67]; sd = 1.56, 95% CI [1.51, 1.61]; AIC = 11176.16), which was a superior fit to the half-normal (sigma = 73.21, 95% CI [65.11, 81.14]; AIC = 12626.58), exponential (rate = 0.026, 95% CI [0.024, 0.029]; AIC = 11,653.53), and power law (alpha = 1.39, 95% CI [1.38, 1.40]; AIC = 11,382.23) distributions (see Figures 1c,d). The lognormal distribution was preferred based on AIC in 999 out of 1,000 bootstrap replicates.

### Transition graph structure after basin size increase

**Figure 2:**
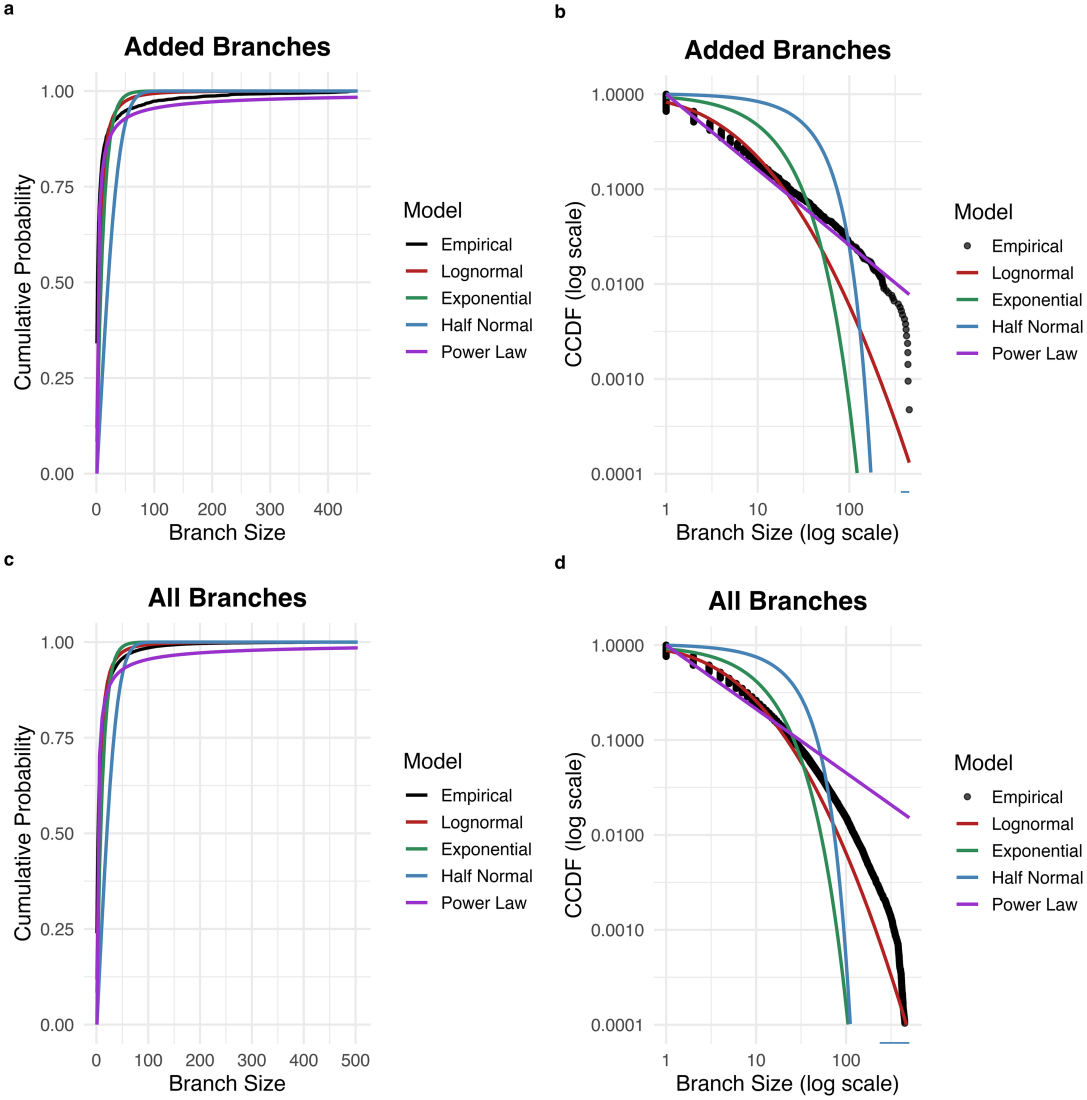
Distribution of branch sizes in network transition graph. **(a)** Cumulative probability distribution for the size of branches added to the target attractor basin. The empirical distribution is best fit by a power law distribution. **(b)** Complementary cumulative distribution function (CCDF) for the size of branches added to the target attractor basin. **(c)** Cumulative probability distribution for the size of all branches across the network. The empirical distribution is best fit by a power law distribution. **(d)** Complementary cumulative distribution function (CCDF) for the size of all branches across the network.

Sizes of branch structures of new states belonging to target attractor basins were computed after increases in basin size. The proportion of branches consisting of a single state was 0.69. Of all branches consisting of two or more states, the estimated excess kurtosis was 50.3, greater than the expected excess kurtosis for a half-normal distribution (~0.87) and for an exponential distribution (Smith and Kounios, 1996). The best fitting distribution was a power law distribution, (AIC = 10501.03), which was a superior fit to the half-normal (AIC = 19,054.53), exponential (AIC = 15,130.96), and lognormal (AIC = 12,508.08) distributions (see Figures 2a,b). The power law fit is further supported by the linear appearance of the empirical distribution on the log–log CCDF plot (with the exception of decreased frequency for the largest branch sizes, which may be explained by truncation of the tail due to the finite size of the network).

For the distribution of branch sizes consisting of two or more states across the entire network, estimated excess kurtosis was 74.0. The best fitting distribution was a power law distribution, (alpha = 1.67, 95% CI [1.67, 1.68]; AIC = 1,389,856), which was a superior fit to the half-normal (sigma = 28.37, 95% CI [27.87, 28.84]; AIC = 1,963,401), exponential (rate = 0.088, 95% CI [0.087, 0.088]; AIC = 1,657,266), and lognormal (mean = 1.49, 95% CI [1.48, 1.49]; sd = 1.25, 95% CI [1.25, 1.26]; AIC = 1,510,063) distributions (see Figures 2c,d). The power law distribution was preferred based on AIC in 1000 out of 1,000 bootstrap replicates.

**Figure 3:**
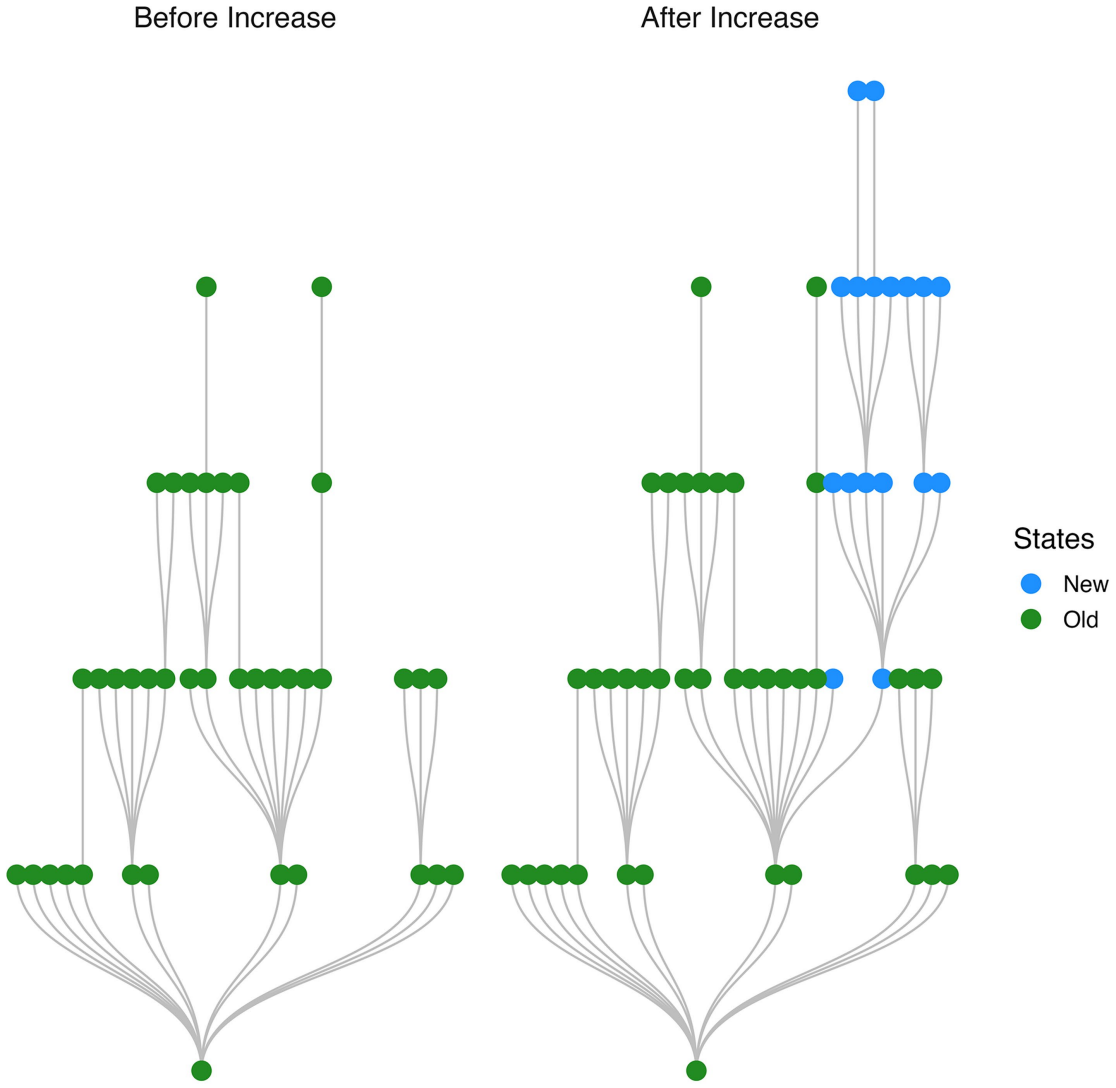
Branching structure of states added to network with a single stimulus presentation. Network transition graph shown immediately before and after a jump in size of the target stimulus attractor basin. Newly added states are organized into a branching structure.

A plot of the graph structure of a target attractor basin immediately before and after a jump in basin size reveals a branching structure of new states (i.e., states which belong to the target basin after the jump but did not belong to the target basin prior to the jump; see Figure 3).

### Sampled initial states in larger networks and effect of interference and stimulus degradation on jump distribution

**Figure 4:**
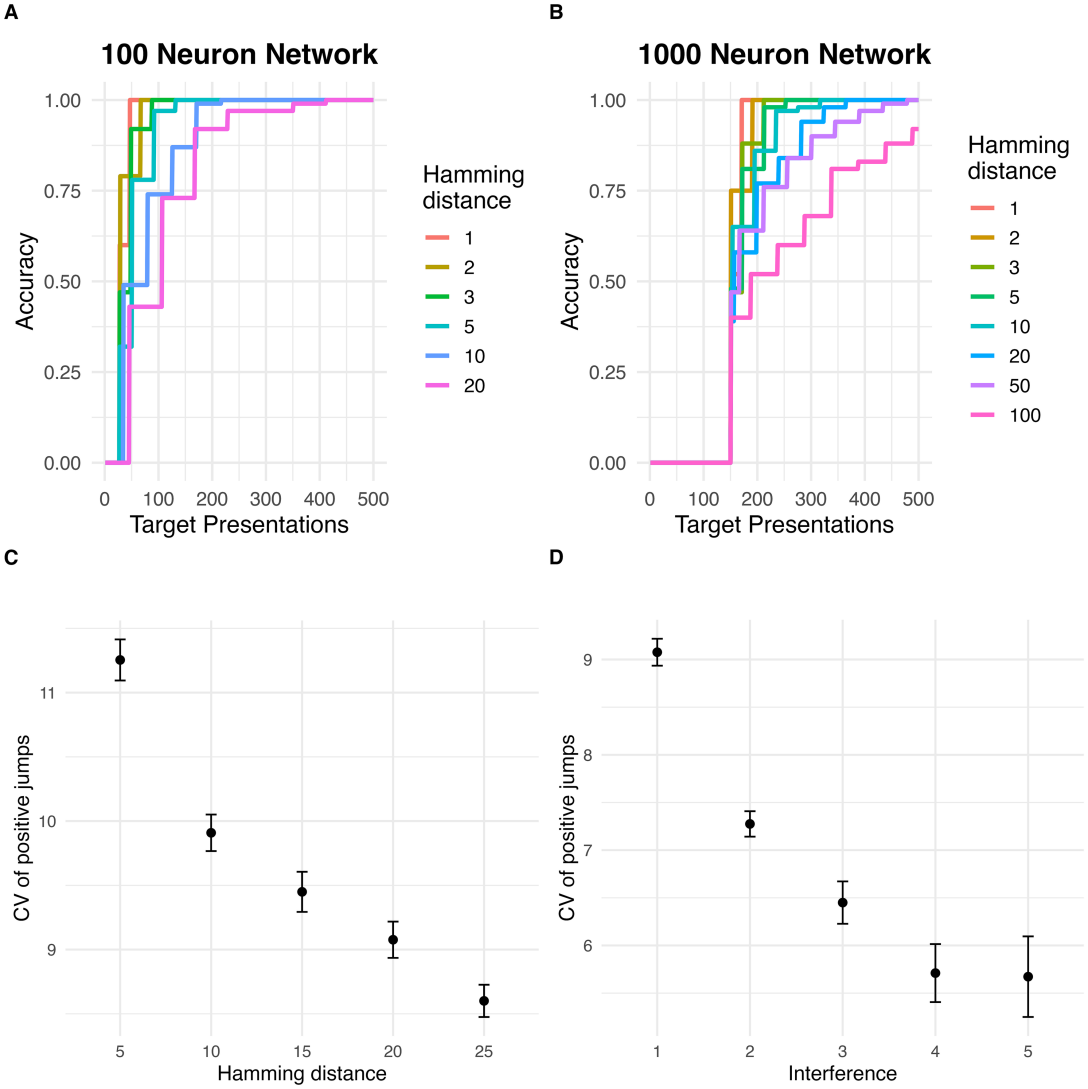
Discrete jumps in accuracy in larger networks. **(A)** Increase in accuracy (percent of states converging to true target) with repeated stimulus presentations for different Hamming distances between target and initial states, in a network with 100 neurons. **(B)** Increase in accuracy (percent of states converging to true target) with repeated stimulus presentations for different Hamming distances between target and initial states, in a network with 1,000 neurons. **(C)** Effect of Hamming distance (i.e., stimulus degradation) on the variability (coefficient of variation) of positive jump sizes. Error bars represent standard error of the mean. **(D)** Effect of number of pretrained memories (i.e., interference) on the variability (coefficient of variation) of positive jump sizes. Error bars represent standard error of the mean.

Similarly to the small networks, large networks displayed evidence of discontinuous jumps in target basin with repeat stimulus presentations. The proportion of sampled initial states at various distances k from the target stimulus which fell within the target basin increased in intermittent, abrupt jumps, rather than gradually and smoothly increasing, in networks with 100 and 1,000 neurons (Figures 4A,B).

A Welch’s ANOVA test revealed that the coefficient of variation of accuracy jump sizes was negatively associated with distance from the target stimulus (i.e., stimulus degradation) (*F* = 184.14, *p* < 0.001; Figure 4C). Similarly, the coefficient of variation of accuracy jump sizes was negatively associated with pretraining stimuli (i.e., memory interference) (*F* = 195.86, p < 0.001; Figure 4D).

## Discussion

In this study, I examined the behavior of Amari-Hopfield networks with a Hebbian update rule with repeated presentations of a single stimulus. I observed that increases in attractor basin size for the target stimulus occurred in abrupt, intermittent bursts, rather than reflecting a smooth, continuous process. I found evidence that the distribution of positive jump sizes in the target attractor basin was heavy-tailed and was best fit by a lognormal distribution. Furthermore, examination of the state transition structure of target attractor basins after jumps revealed that newly acquired states were frequently organized into branching structures, with the size of these branches being best approximated by a power law distribution. These findings are consistent with the hypothesis that the heavy-tailed distribution of jumps in the size of the target basin size may be related to the heavy-tailed distribution of branch sizes in the network state transition graph, although these distributions are not identical. Additionally, both stimulus degradation (Hamming distance between start state and target) and memory interference (number of pretrained memories) were negatively associated with variability in jump sizes, establishing qualitative predictions for future behavioral studies of discrete changes in performance.

Amari-Hopfield networks represent high-level, idealized models which may be relevant to the implementation of associative memory in recurrent neural networks (RNNs) in the brain. The effects observed here, i.e., threshold effects with gradual parameter changes, may also be observed in more complex, biologically plausible network models. Such models, which may incorporate graded or spiking activation, stochasticity and noise, more complex learning rules (such as spike timing dependent plasticity [Caporale and Dan, 2008)], complex (rather than full) connectivity patterns, interneuron populations, and other features of biological networks may thus be investigated to better understand threshold effects which are clearly illustrated in the simpler Amari-Hopfield model. Investigation of biologically realistic mechanisms, such as spike timing dependent plasticity, may also help to elucidate the role of precise timing in behavioral paradigms, as well as the effect of specific neuromodulatory influences on synaptic learning mechanisms, in altering jumps in performance. Continuous, spiking recurrent networks with spike timing depending plasticity mechanisms have been investigated in detail (Seeholzer et al., 2019; Mongillo et al., 2008; Amit and Brunel, 1997), and future studies can examine the effect of repeat stimulus presentations in similar networks. The approach may also be extended by incorporating additional biological mechanisms such as stimulus specific adaptation, in which neural responses to common stimuli are suppressed (Nelken, 2014). Hebbian learning, as studied here, may underpin the recognition of common stimuli, contributing to the suppression of response to those stimuli in perceptual systems. Adaptation itself may also have complex effects on recurrent network performance (Cortes et al., 2012). The current results may thus be a stepping stone to investigation of more complex phenomena in biological systems. Similarly, characterization of basic phenomena in simple nonlinear models has previously helped guide more detailed, realistic studies of weather and climate (Lorenz, 1991), population dynamics (Kendall and Fox, 1998), and other complex systems. In addition to extension to more detailed neural network models, the results can also be extended to more detailed cognitive models of particular learning domains. While it is likely that the specific details of discontinuous changes in performance with learning in a given domain [e.g., language learning (van Dijk and Geert, 2007)] are influenced by domain-specific factors, the ubiquity of these effects across domains (Donner and Hardy, 2015; Smith and Kounios, 1996; Musfeld et al., 2023) suggests there may be a common mechanism related to associative learning in nonlinear networks.

The current results are consistent with the hypothesis that the branching state transition structure of attractor basins in the Amari-Hopfield network are related to the discontinuous changes in attractor basin size with repeated stimulus presentations. The branch size, i.e., number of descendant nodes, across all states in the network is best approximated by a power law distribution, as are the branch sizes of newly acquired states after a jump in attractor basin size. The distribution of increases in attractor basin size also appears to be heavy-tailed, although best fit by a lognormal rather than power law distribution. The difference in distributions between jump sizes and branch structures is likely explained by the fact that more than one branch can be added to the attractor basin in a single step, including a relatively large number of single states. A full theoretical analysis of the observed discontinuous changes in attractor size is beyond the scope of this brief report. Future work can apply theoretical techniques such as mean field approximations to improve understanding of the observed behaviors.

The results highlight the importance of examining individual, in addition to average, behavior to develop models of the mechanistic underpinnings of cognitive performance. Fitting average rather than individual data can obscure the true generating process, especially when this process is nonlinear (Busemeyer and Diederich, 2010). Thus, while many studies of learning report smooth acquisition curves when behavior is averaged at the group level, studies examining individual behavior often report discontinuous, step-like acquisition (Musfeld et al., 2023). This distinction between individual and group learning curves mirrors the current results, in which a single run of the Amari-Hopfield network displays discontinuous jumps (Figure 1a), while an average of multiple runs appears to show a smooth expansion in basin size (Figure 1b). Employing an individual-differences approach can help constrain and validate cognitive theories (Vogel and Awh, 2008). Fitting models to individual vs. group data represents a tradeoff, with consideration of individual data being necessary to maximize accuracy (Estes and Todd Maddox, 2005). Bayesian hierarchical approaches have become an increasingly popular tool to mitigate the tradeoff between group and individual fitting (Scheibehenne and Pachur, 2015).

Future research can further investigate individual differences by examining the effect of network parameters (e.g., representing neuromodulatory influences or individual differences in network wiring) on the distribution of jumps and the transition graph structure. Another limitation is that the analyses were limited to classical Amari-Hopfield networks with a traditional Hebbian learning rule. Future research can also examine jump behavior in the context of alternative network architectures or learning rules, such as dense associative memories (Krotov and Hopfield, 2016). Additionally, the current results pertain to deterministic networks. Future research can examine these effects in stochastic associative networks (Pantic et al., 2002). These studies can examine whether alternative network structures evidence different distributions of jump sizes and branch sizes. Another interesting question is under what circumstances the phenomenon of discrete jumps in performance in human memory is adaptive (e.g., by facilitating rapid memory consolidation with stimulus exposure) or maladaptive (e.g., by destabilizing performance).

In sum, the present results provide evidence that even the simplest network models of associative learning can exhibit sudden, discontinuous jumps in learning performance. Similar threshold effects, in which small input changes can trigger large alterations in attractor structure, may also occur in biological RNNs and could have important implications for cognitive functions such as declarative and procedural memory. Future work using more biologically plausible neural models is warranted to further explore these dynamics and to develop more detailed accounts of the mechanisms underlying discontinuous learning in humans.

## Data Availability

The datasets presented in this study can be found in online repositories. The names of the repository/repositories and accession number(s) can be found at: https://github.com/jonhowlett/hopfield-jumps.
